# Role of Conditioning and Verbal Suggestion in Placebo and Nocebo Effects on Itch

**DOI:** 10.1371/journal.pone.0091727

**Published:** 2014-03-19

**Authors:** Danielle J. P. Bartels, Antoinette I. M. van Laarhoven, Elise A. Haverkamp, Oliver H. Wilder-Smith, A. Rogier T. Donders, Henriët van Middendorp, Peter C. M. van de Kerkhof, Andrea W. M. Evers

**Affiliations:** 1 Unit of Health, Medical and Neuropsychology, Institute of Psychology, Leiden University, Leiden, The Netherlands; 2 Department of Medical Psychology, Radboud University Medical Center, Nijmegen, The Netherlands; 3 Department of Anesthesiology, Pain, and Palliative Medicine, Radboud University Medical Center, Nijmegen, The Netherlands; 4 Department for Health Evidence, Radboud University Medical Center, Nijmegen, The Netherlands; 5 Department of Dermatology, Radboud University Medical Center, Nijmegen, The Netherlands; Tokai University, Japan

## Abstract

Placebo and nocebo effects are known to play a key role in treatment effects in a wide variety of conditions. These effects have frequently been investigated with regard to pain and also in other physical sensations, but have hardly been investigated with regard to itch. In addition, neither in pain nor in any other physical sensation, the single and combined contribution of the expectancy mechanisms of conditioning and verbal suggestion have ever been investigated in both placebo and nocebo effects within one design. For the first time, the role of verbal suggestion and conditioning in placebo and nocebo effects on itch was experimentally investigated. Expectations about itch stimuli were induced in healthy subjects by verbal suggestion, conditioning, or a combination of both procedures, and compared with a control group without expectation induction. Itch was induced electrically by means of quantitative sensory testing. Significant placebo and nocebo effects were induced in the group in which combined procedures of conditioning and verbal suggestion were applied in comparison with the control group. The conditioning and verbal suggestion procedures applied individually did not induce significant placebo and nocebo effects when compared with the control group. The results of this study extend existing evidence on different physical sensations, like pain, by showing that also for itch, the combination of conditioning and verbal suggestion is most promising in inducing both placebo and nocebo effects. More research on placebo and nocebo effects at a perceptive and neurobiological level is warranted to further elucidate the common and specific mechanisms underlying placebo and nocebo effects on itch and other physical sensations.

## Introduction

Placebo and nocebo effects are treatment effects, unrelated to the treatment mechanism, which are induced by patients' expectations of improvement or worsening respectively [1]–[3]. Placebo and nocebo effects are known to contribute to the outcome of treatment effects for a range of symptoms and conditions like Parkinson's disease, gastrointestinal disorders, nausea, fatigue and pain [1], [3]–[8]. In contrast to the extensive placebo research mainly on pain, hardly any placebo research has focused on itch, which is a common symptom of several conditions and diseases, such as dermatological and systemic diseases, and can be a considerable burden to patients especially when symptoms are chronic [9], [10]. Moreover, itch particularly seems highly susceptible to suggestion, as demonstrated by the phenomenon of “contagious” itch: e.g., watching someone scratch himself can induce a sensation of itch in the perceiver (e.g., [11], [12]). Therefore, placebo and nocebo effects might be relevant to itch in particular.

Mechanisms underlying placebo and nocebo effects have extensively been investigated, especially in the field of pain. Expectation induction mechanisms of verbal suggestion and conditioning have been identified as central processes eliciting placebo and nocebo effects, by decreasing or increasing symptoms respectively, when administering an inert (placebo) treatment or agent [2], [13], [14]. With regard to pain, verbal suggestion has been shown to induce short-term nocebo effects, whereas conditioning is particularly relevant to induce placebo effects and more robust nocebo effects [13], [15], [16]. Also in other physical sensations such as fatigue and nausea conditioning seems to be particularly relevant [5], [8], [17]. With regard to itch, the role of conditioning in placebo or nocebo effects has not been investigated yet, although, there is some evidence for the role of verbal suggestion in placebo and nocebo effects on itch. For example, patients with atopic dermatitis react more strongly to histamine after nocebo-related itch suggestions [18], and in a previous experiment, we showed that verbal suggestion alone can induce nocebo and possibly also placebo effects on itch [19].

Most studies investigating the role of conditioning in placebo and nocebo effects applied conditioning in combination with verbal suggestion. The few studies that used a conditioning procedure without verbal suggestion yielded mixed results [20]–[23]. Hardly any research has compared the single and combined contributions of verbal suggestion and conditioning to placebo effects. Moreover, to the best of our knowledge, no direct comparison of verbal suggestion, conditioning, and the combination of both has been made yet with regard to nocebo effects within one design.

The aim of this study was to investigate the role of verbal suggestion and conditioning in both placebo and nocebo effects on itch. Alike pain and other physical sensations, it was hypothesized that the expectation induction, particularly the combination of conditioning and verbal suggestion, would result in decreased (placebo) and increased (nocebo) itch in comparison to a control procedure. In addition, it was explored whether individual characteristics related to negative (e.g., neuroticism) or positive (e.g., optimism) outcome expectancies were associated with individual placebo and nocebo responses [13], [24]–[27].

## Methods

### Ethics statement

The study was approved by the regional medical ethics committee CMO regio Arnhem-Nijmegen and follows the rules stated in the Declaration of Helsinki. All participants gave written informed consent and were reimbursed for their participation.

### Participants and general procedure

Healthy subjects were recruited at the campus of the Radboud University Nijmegen, Nijmegen, the Netherlands. Exclusion criteria were severe morbidity (e.g., skin disease, multiple sclerosis, diabetes mellitus), psychiatric disorders (e.g., depression), color blindness, regular use of medication in the last 3 months, use of pacemaker, and current or past chronic itch or pain. Participants were told that the purpose of the study was to determine sensitivity to itch stimuli. At least one week prior to the experiment, a session took place in which previous experiences and expectations of sensations such as itch and pain of all subjects were assessed (results not reported here). In addition, subjects were sent self-report questionnaires about individual characteristics to be completed at home. As one subject unexpectedly went abroad after the first session, 95 subjects completed the experiment. All 95 subjects were of Dutch nationality, and were aged 18 years or older (mean age 22.7±3.2 years); 77% were women. Of the subjects, 54% had a partner (13% married or living with a partner) and 58% used hormonal contraceptives. On the test day, the mean baseline levels of itch and pain were 0.5 (SD = 0.8) and 0.5 (SD = 0.7), respectively, as rated on a visual analogue scale (VAS) ranging from 0 (no itch/pain at all) to 10 (worst itch/pain ever experienced).

### Test day procedure

**Figure 1:**
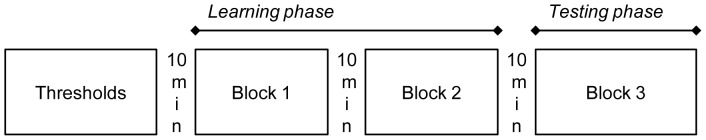
Flow diagram showing the experimental procedures of the study in chronological order.

For the test day, all subjects were asked to refrain from drinking coffee, tea, or energy drink from one hour before testing, which took place at a fixed time in the afternoon. A schematic overview of the study is displayed in Figure 1. At first, all subjects held their hands in a warm water bath at about 32°C for 3 minutes [28], in order to attain a comparable baseline wrist skin temperature among participants. Then the itch thresholds were determined by gradually increasing the intensity of the electric current with a ramping procedure (see *Methods* *; Itch induction*). Thereafter, subjects were randomly assigned to one of four groups, using a computer generated randomization list. Since the instructions given to the subjects differed in accordance with the group the subjects were allocated to, only subjects were blinded for the randomization to different groups. In line with previous conditioning studies of nocebo and placebo effects on pain [15], [16], the experimental session comprised two phases: a learning phase and a testing phase. In both phases, itch stimuli were preceded by visual cues (colored lights, i.e., green, yellow, and red lights) displayed on a computer screen. The learning phase consisted of two blocks, in which either no expectations were induced or participants received verbal suggestion, conditioning, or a combination of both procedures, to induce expectations about the intensity of the itch stimuli. In this phase, itch stimuli of a varying intensity were applied, preceded by a cue (6x green, 6x yellow, and 6x red cue). In the testing phase, itch stimuli were all applied at medium intensity, preceded by a cue (5x green, 5x yellow, and 5x red cue). After the threshold measurements and in-between the different experimental blocks, there was a standardized 10-minute break in which participants were provided with a selected number of magazines to read with a neutral content (about nature and home decoration), and they were offered a small snack and herbal tea or water (see also Fig. 1)

### Experimental groups and control group

**Figure 2:**
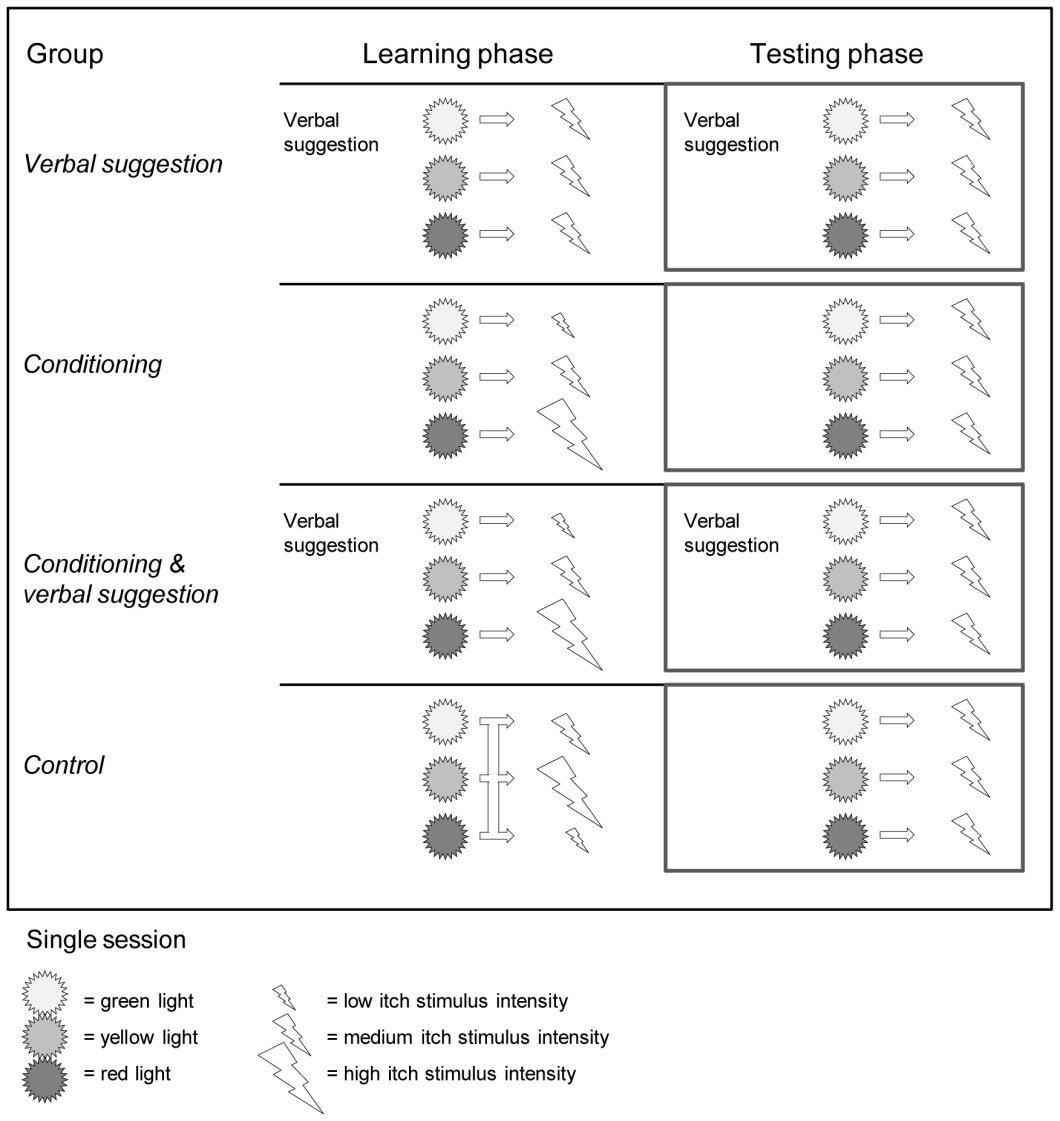
Experimental design. Subjects were randomly assigned to one of four groups: verbal suggestion; conditioning; verbal suggestion with conditioning; and control. In the learning phase verbal suggestion and conditioning procedures depended on the experimental group. In the testing phase the verbal suggestion was in correspondence with the verbal suggestion applied in the learning phase, while all participants received itch stimuli of a medium intensity.

The experimental design is displayed in Figure 2. In the *verbal suggestion group*, expectations of low, neutral, and high levels of itch were raised in subjects by telling them that different cues (colored lights on the computer screen) indicated that the stimulus intensity would be altered. This change would be brought about by a third electrode, which was actually a placebo or sham electrode (inactive electrode): “A green light will signal the activation of the third electrode that induces a decrease in the intensity of the itch stimulus. A red light will signal an increase in the intensity of the itch stimulus by the activation of the electrode, and the yellow light will indicate that the third electrode is turned off and will not change the intensity of the itch stimulus”. Regardless of the color of the cue displayed, all stimuli had a medium intensity. In the *conditioning group*, expectations of low, neutral, and high levels of itch were raised in subjects by the repeated pairing of the green, yellow, and red cues with low, medium, and high itch stimulus intensities, respectively. The current intensities (mA) for the low, medium, and high stimulus intensities were determined according to the participants' individual itch thresholds (see *Methods* *; itch induction*). No verbal suggestion was given to avoid any verbal suggestion effects, i.e., subjects were not given information about the stimulus intensity, but were merely told that several itch stimuli would be applied after the presentation of color cues. In the *conditioning with verbal suggestion group*, the conditioning procedure and the verbal suggestion procedure were combined, thus applying stimuli of low, medium, and high intensity concurrently with the green, yellow, and red cues, respectively, and the corresponding verbal suggestion. In the *control group*, no expectations regarding the itch stimuli were induced, neither by verbal suggestion nor by conditioning, i.e., subjects were not given information about the colored cues or stimulus intensity, and itch stimuli were given independently of the colored cue at a predetermined random order at low, medium, and high intensity. Unlike in the learning phase, in the testing phase only stimuli of medium intensity were applied in all groups. The verbal suggestion given in the testing phase corresponded with the verbal suggestion given in the learning phase (See Fig. 2. for the experimental design).

### Itch induction

Itch was induced by means of electrical stimulation by a constant current stimulator (Isolated Bipolar Constant Current Stimulator DS5, Digitimer, United Kingdom), and delivered to the inner side of the non-dominant wrist through two surface electrodes (a disk electrode of ø 1 cm and a reference electrode of ø 2 cm, VCM Medical, the Netherlands). The stimulator was coupled to a data acquisition system (NI-DAQmx, National Instruments, Hungary), which was controlled by a laptop. One electrode was applied 1.5 cm proximal to the triquetrum, at the center of the inner wrist, while the reference electrode was applied 2 cm below. A third (sham) electrode was placed about 1 cm left from the two real electrodes and attached to the back of the stimulator. Stimuli were applied at 50-Hz frequency with a pulse duration of 100 µs [29] and at a continuously increasing current intensity (0.05 mA/s) up to a maximum current intensity of 5 mA. After each stimulus, participants were asked to report the level of itch on a visual analogue scale (VAS) ranging from 0 (no itch at all) to 10 (worst itch ever experienced). The following thresholds were measured three times by gradually increasing the current intensity from 0 mA up to the intensity at which the respective threshold had been reached: “the first moment you feel some itch” (IT1); “the first moment you feel the urge to scratch” (IT2); and “the first moment you cannot resist the urge to scratch” (IT3). The mean of these thresholds was used for the calculation of the individual current intensities of the low, medium, and high itch stimulus applied in the experimental phase. In-between every stimulus applied in the learning and testing phase, there was a 2-minute interval, in which filler tasks (e.g., puzzles) were given to diminish possible influence of itch evoked by previously applied stimuli on subsequent stimuli. The interval could be extended to a maximum of 4 minutes if the level of itch after 2 minutes was ≥2 on a VAS.

### Questionnaires

Individual psychological characteristics of optimism, hope, neuroticism, extraversion, negative affect and worrying were assessed by means of self-report questionnaires, previously shown to have satisfactory reliability and validity.

### Optimism

The Revised Life Orientation Test (LOT-R) [30] was used to measure optimism, the tendency to expect positive outcomes. The LOT-R consists of 10 items scored on a 5-point Likert scale ranging from 0 (“Strongly Disagree”) to 4 (“Strongly Agree”). Total scores range from 0 to 24, with higher scores indicating higher levels of dispositional optimism. In the present study, Cronbach's alpha was 0.79.

### Hope

The Dispositional Hope Scale (DHS) was used to measure hope, the tendency to experience a reciprocally derived sense of successful agency and pathways [31], [32]. The DHS consist of 12 items scored on a 8-point Likert scale ranging from 1 (“Definitely False”) to 8 (“Definitely true”), with higher scores indicating higher levels of hope. In the present study, Cronbach's alpha was 0.78.

### Neuroticism & Extraversion

The Eysenck Personality Questionnaire (EPQ) was used to measure neuroticism, the tendency to experience more negative affect and negative outcome expectations, and extraversion, the tendency of having more outgoing, talkative, and energetic behavior [33]. The neuroticism and extraversion subscales consist of 22 and 19 “yes/no” items, respectively. Higher scores indicate higher levels of neuroticism and extraversion. In the present study, Cronbach's alpha was 0.84 for neuroticism and 0.86 for extraversion.

### Negative affect

The Hospital Anxiety and Depression Scale (HADS) was used to measure negative affect characterized by symptoms of depression and anxiety [34]. The HADS consists of 14 items scored on a 4-point Likert scale ranging from 0 (“no problem”) to 3 (“severe problem”), with higher scores indicating higher levels of negative affect. In the present study, Cronbach's alpha was 0.80.

### Worrying

The Penn State Worry Questionnaire (PSWQ) [35] was used to measure worrying, which includes the tendency to experience more negative outcome expectancies. The PSWQ consists of 16 items scored on a 5-point Likert scale, ranging from 1 (“not at all typical of me”) to 5 (“very typical of me”), with higher scores indicating greater worrying. In the present study, Cronbach's alpha was 0.93.

### Statistical analysis

All analyses were performed using SPSS 20.0 for Windows (SPSS Inc. Chicago, Illinois, USA). Analyses of variance (ANOVA) and Chi-square tests were used to test for baseline differences in demographic variables between the four groups. Means of the VAS itch scores were calculated for the learning and testing phases in all groups. Variables were checked for outliers and skewness as these can severely limit the usefulness of the mean as measure for location. Since there was no indication of problems in this respect, untransformed variables were analyzed. In order to be able to measure nocebo and placebo effects, i.e., by an increase or a decrease in itch respectively, an intermediate itch intensity was introduced by applying a stimulus at medium intensity preceded by a yellow cue along with a neutral expectation. The nocebo effect was then defined as the difference between the mean itch VAS scores associated with the five red cues and the five yellow cues in the testing phase, and the placebo effect was defined as the difference between the mean itch VAS scores associated with the five green cues and the five yellow cues in the testing phase. Univariate analyses of variance (ANOVAs) were performed with *group* as between-subject factor for nocebo and placebo effects, in order to test the hypothesis, i.e., that the experimental groups would display significant nocebo and placebo effects in comparison with the *control group*. Post hoc Dunnett tests were conducted to compare the experimental groups separately with the *control group*. The effectiveness of the expectation induction procedures was also exploratively assessed during the learning phase. Again, separate ANOVAs and post hoc Dunnett tests were performed as described above, exploring the difference in itch VAS scores between the green- or red- and yellow-associated stimuli in the learning phase. Exploratively, in the three experimental groups Pearson correlation coefficients were calculated between the nocebo and placebo effects and questionnaire scores for individual characteristics. For all analyses, the level of statistical significance was set at *p*<0.05.

## Results

### Experimental and control groups

Randomization of the subjects across the different experimental and control groups resulted in a total of 23 subjects in the *verbal suggestion group*, 24 subjects in the *conditioning group*, 23 subjects in the *conditioning with verbal suggestion group*, and 25 subjects in the *control group*. There were no significant between-group differences with regard to age, gender, use of hormonal contraceptives, and baseline levels of itch and pain on the test day.

### Nocebo effects

#### Learning phase for induction of nocebo effects

Table 1 displays the mean (±SD) itch VAS scores evoked by the stimuli associated with the red and yellow cues during the learning phase for the four groups. When exploring whether the difference in the levels of electrically evoked itch (i.e., red minus yellow cue) would be larger in the three experimental groups than in the *control group*, Univariate analysis of variance (ANOVA) revealed a significant between group effect (*F*(3,91) = 49.528, *p*<0.001). Post hoc Dunnett tests indicated a significantly larger itch VAS difference score between the red- and yellow-associated stimuli, for the *verbal suggestion group* (*p*<0.001), the *conditioning group* (*p*<0.001) and the *conditioning with verbal suggestion group* (*p*<0.001) as compared with the *control group*.

**Table 1:** Means and standard deviations for itch VAS scores in the learning phase for the different groups.

	Itch VAS scores (M ± SD)		
Group	Green cue	Yellow cue	Red cue
*Verbal suggestion*	4.56±1.81	4.77±1.78	5.39±1.83
*Conditioning*	3.73±2.07	4.54±2.09	5.84±2.01
*Conditioning & Verbal suggestion*	2.37±1.75	3.97±1.34	6.04±1.55
*Control*	3.52±2.00	3.43±2.01	2.87±1.68

Means (M) and standard deviations (SD) of the visual analogue scale (VAS) scores for itch in the verbal suggestion group (*n = 23*), conditioning group (*n = 24*), conditioning with verbal suggestion group (*n = 23*) and control group (*n = 25*) in the learning phase.

#### Testing phase nocebo effects

**Figure 3:**
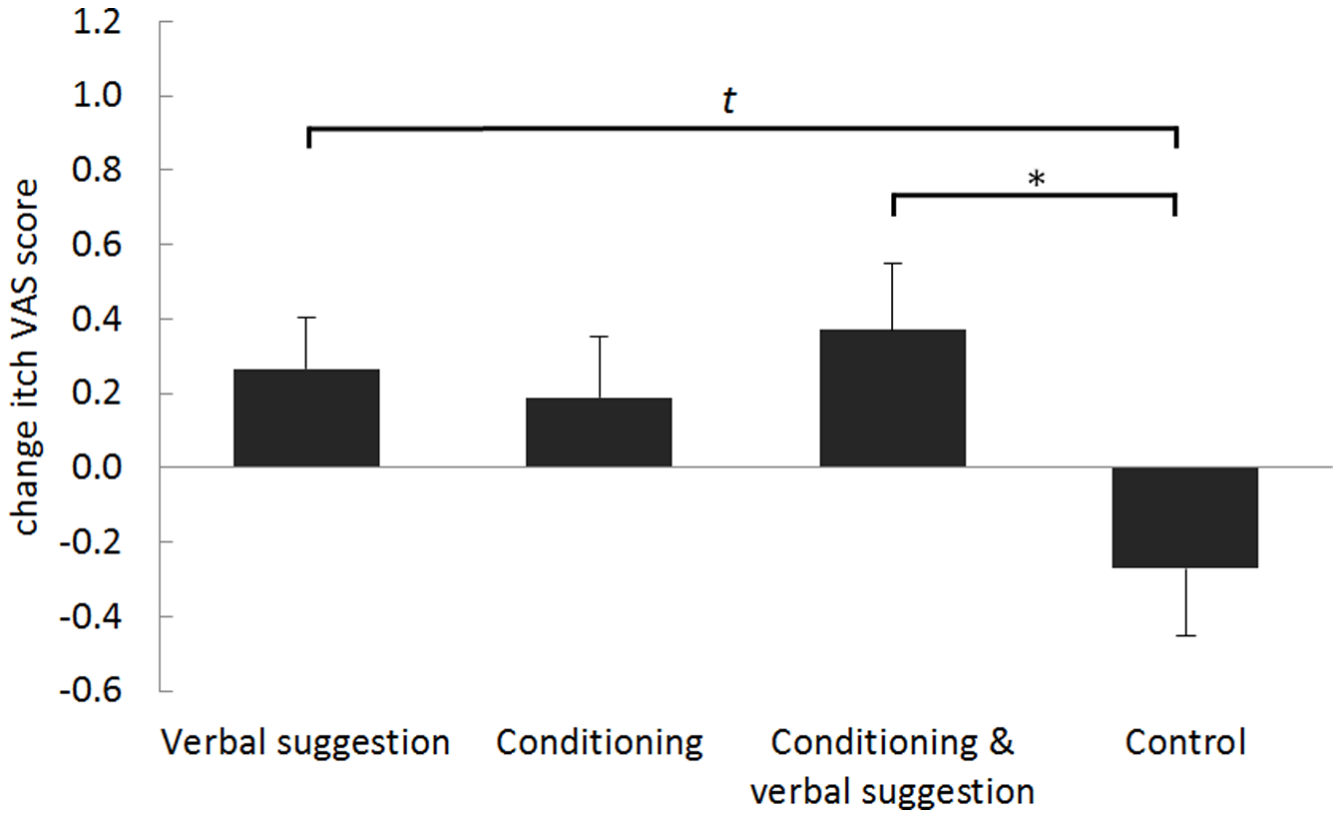
Means and standard error of the mean of the visual analogue scale (VAS) itch scores for the nocebo effect (change VAS score between the red and yellow cues) of the four groups in the testing phase. The asterisks show the level of significance related to the post hoc Dunnett comparison of the nocebo effect between the experimental groups and the control group (**p<0.01; *p<0.05; *t* = *p*<0.10).

Table 2 displays the mean (±SD) itch VAS scores evoked by the stimuli associated with the red and yellow cues during the testing phase for each group (in which all stimuli were applied at medium intensity), and the mean nocebo effect for each group is shown in Figure 3. Univariate ANOVA showed a significant difference in the magnitude of the nocebo effect in the different groups (*F*(3,91) = 2.995, *p* = 0.035). Post hoc Dunnett tests comparing the experimental groups with the *control group* indicated a significant nocebo effect in the *conditioning with verbal suggestion group* (*p* = 0.020), a borderline significant nocebo effect in the *verbal suggestion group* (*p* = 0.063), and no significant nocebo effect in the *conditioning group* when compared with the *control group* (See Fig. 3.).

**Table 2:** Means and standard deviations for itch VAS scores in the testing phase for the different groups.

	Itch VAS scores (M ± SD)		
Group	Green cue	Yellow cue	Red cue
*Verbal suggestion*	3.20±1.91	3.60±1.91	3.87±2.05
*Conditioning*	3.30±1.87	3.41±1.80	3.59±1.87
*Conditioning & Verbal suggestion*	2.42±1.68	3.28±1.71	3.65±2.00
*Control*	2.33±1.62	2.65±1.87	2.38±1.70

Means (M) and standard deviations (SD) of the visual analogue scale (VAS) scores for itch in the verbal suggestion group (*n = 23*), conditioning group (*n = 24*), conditioning with verbal suggestion group (*n = 23*), and control group (*n = 25*) in the testing phase.

### Placebo effects

#### Learning phase for induction of placebo effects

Table 1 displays the means and standard deviations (SD) of the itch VAS scores evoked by the stimuli associated with the green and yellow cues during the learning phase for the four groups. When exploring whether the difference in the levels of electrically evoked itch (i.e., yellow minus green cue) would be larger in the three experimental groups than in the *control group*, Univariate analysis of variance (ANOVA) revealed a significant group effect (*F*(3,91) = 16.742, *p*<0.001). Post hoc Dunnett tests indicated a significantly larger itch VAS difference score between the green- and yellow-associated stimuli, for the *conditioning group* (*p* = 0.002) and the *conditioning with verbal suggestion group* (*p*<0.001), as compared with the *control group*. In the comparison to the *control group*, the *verbal suggestion group* did not differ in itch VAS difference score between the green- and yellow-associated stimuli.

#### Testing phase placebo effects

**Figure 4:**
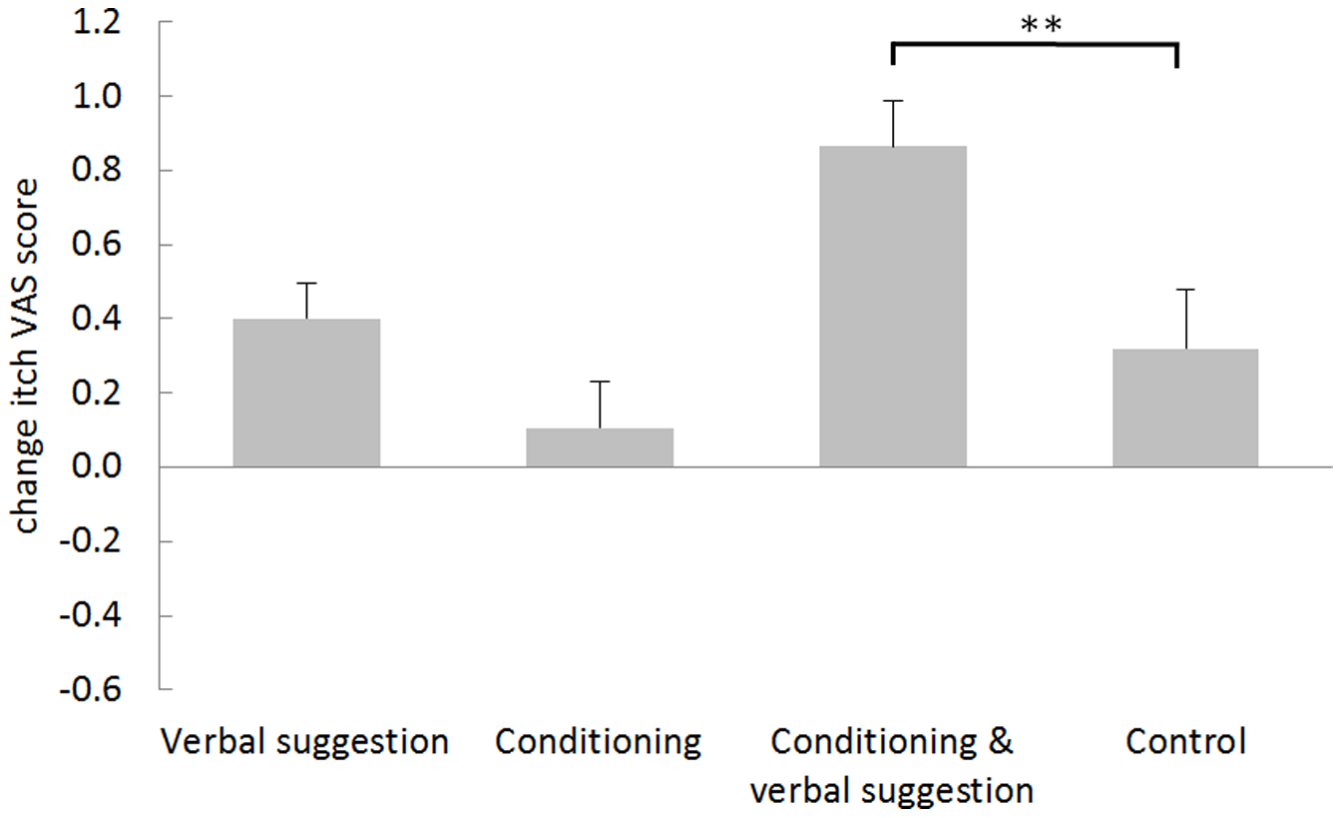
Means and standard error of the mean of the visual analogue scale (VAS) itch scores for the placebo effect (change VAS score between the green and yellow cues) of the four groups in the testing phase. The asterisks show the level of significance related to the post hoc Dunnett comparison of the placebo effect between the experimental groups and the control group (**p<0.01; *p<0.05; *t* = *p*<0.10).

Table 2 displays the mean (±SD) itch VAS scores evoked by the stimuli associated with the green and yellow cues during the testing phase for each group (in which all stimuli were applied at medium intensity), and the mean placebo effect for each group is shown in Figure 4. Univariate ANOVA showed a significant difference in the magnitude of the placebo effect in the different groups (*F* (3,91) = 6.154, *p* = 0.001). Post hoc Dunnett tests comparing the experimental groups with the *control group* indicated a significant placebo effect in the *conditioning with verbal suggestion group* (*p* = 0.009), but no significant differences in the placebo effect were found in the *verbal suggestion group* or the *conditioning group* when compared with the *control group* (See Fig. 4.).

### Individual characteristics

#### Nocebo effect

In the *conditioning with verbal suggestion group*, significant correlation coefficients were found between a greater nocebo response and more worrying (*r* = 0.485; *p* = 0.019), more negative affect (*r* = 0.433; *p* = 0.039), less hope (*r* = −0.452; *p* = 0.030), and lower levels of extraversion (*r* = −0.511; *p* = 0.013), but not with neuroticism or optimism. No significant correlations were found in the other experimental groups.

#### Placebo effect

The magnitude of the placebo effect was not significantly correlated with the individual characteristics of optimism, hope, extraversion, neuroticism, negative affect, and worrying in any of the three experimental groups, except for a significant correlation coefficient between a greater placebo effect and less hope in the *conditioning with verbal suggestion group* (*r* = −0.507; *p* = 0.014).

## Discussion

For the first time, expectation induction procedures of verbal suggestion, conditioning and the combination of both were investigated with regard to both nocebo and placebo effects on itch. Results show that nocebo and placebo effects can be induced on itch, with the strongest effects elicited by a combination of conditioning and verbal suggestion rather than by either procedure alone. These results are in line with research in nocebo and placebo effects on pain and other physical sensations such as nausea and fatigue (e.g., [5], [8], [13], [15]).

The subjects receiving both conditioning and verbal suggestion procedures had significantly higher (nocebo) and lower (placebo) levels of induced itch than did the subjects receiving the control procedure. Additional support for these findings was found in the learning phase, during which there was a similar pattern of changes in itch VAS scores in response to nocebo and placebo cues. These findings are consistent with earlier research on pain which showed that the combination of conditioning and verbal suggestion evoked robust hyperalgesic and analgesic effects [13], [15], [36]. Moreover, when verbal suggestion procedures were combined with conditioning, generally larger and longer-lasting nocebo and placebo effects could be induced when compared with these procedures alone [13], [15], [20], [22], [23], [36], [37]. In addition, also in other physical symptoms further support has been found for the combination of conditioning and verbal suggestion such as in nausea and fatigue [5], [38]. For inducing nocebo and placebo effects on various physical sensations, the combination of these expectation induction mechanisms seems most promising.

Verbal suggestion (without conditioning) elicited a marginally significant nocebo effect on itch when compared with a control procedure during the testing phase, and elicited a robust significant nocebo effect in the learning phase. Previous research has shown that robust nocebo effects on itch [19] and pain [1], [2], [13], [14] can be induced by verbal suggestion. In contrast, placebo effects were not significantly induced by verbal suggestion when compared with a control procedure, i.e., subjects did not experience less itch when told that the stimulus would evoke less itch, than when given neutral suggestions (control group). However, indirect indications were found for a placebo effect within the verbal suggestion group by showing that these subjects experienced significantly lower levels of itch for the stimuli associated with the green (placebo) versus the yellow (neutral) cues (see table 2). Using two phases (learning and testing phase) might have led to extinction of effects that were present in the first (learning) phase, while to test for the effects of verbal suggestion a single phase is sufficient [15].

Conditioning (without verbal suggestion) did not elicit significant nocebo or placebo effects on itch in the testing phase (in which all itch stimuli were of the same intensity). This finding was somewhat unexpected since significant altered itch scores were present in the learning phase. A reason for non-significant effects of conditioning might be the number or length of learning trials. The few previous studies that investigated the effects of conditioning (without verbal suggestion) on pain generally found significant nocebo or placebo effects for example when more or longer lasting learning trials were used [20]–[23]. During conditioning, the perception of an increase or decrease in sensations after a cue can shape both automatic and conscious expectations about the given cue [39], and with more or longer conditioning trials, the learned association may be more predictable. Moreover, the addition of explicit expectations (by verbal suggestion) might further amplify the induction of nocebo and placebo effects on physical sensations, and itch in particular which seems highly susceptible to suggestion as demonstrated by the phenomenon of “contagious” itch (e.g., [11], [12]).

Individual characteristics related to outcome expectancies were, in contrast to the placebo effect, associated with the nocebo effect (more negative affect, less extraversion, more neuroticism and less hope). This is in line with previous findings on nocebo effects [13], [24]–[27], and studies showing that particularly negative affect and cognitions can enhance itch [19], [40]–[43]. These findings also support the idea that negative (aversive responses) rather than positive (safety responses) expectations may be easier to induce from an evolutionary perspective in order to promote survival [15], [44], possibly mediated by a tendency to experience more negative affect and cognitions, such as worrying. These findings also underline the possibly divergent mechanisms underlying nocebo and placebo effects. While anxiety or stress-related processes are thought to be involved in nocebo hyperalgesia, e.g., through an increase in cortisol plasma concentrations, reward processes are supposed to be involved in placebo analgesia, e.g., by the activation of dopamine neurotransmission [39], [45]. Additional research into the possible predictors and different mechanisms of nocebo and placebo responding is clearly required.

Some limitations and implications for future research should be discussed. First, the number of conditioning trials in the learning phase might have been insufficient to induce nocebo or placebo effects in the group receiving conditioning alone (i.e., without verbal suggestion) [20]–[22]. Second, placebo effects were not only found in the experimental groups, as expected, but also in the control group there was a tendency for a decrease in itch (see table 2). The expectations of the participants regarding the visual stimuli may have unintentionally been influenced because, during the random allocation of cues to a stimulus intensity, the yellow cue was more often (than by chance) associated with a high intensity itch stimulus. This could, for example, explain the lack of a significant placebo effect in the *verbal suggestion group* in relation to the *control group*. Third, in contrast to previous studies investigating the role of either negative or positive expectations on itch [18], [19], [46], in this study we induced nocebo and placebo expectations at the same time in individual participants. Since different mechanisms may underlie the induction of nocebo and placebo effects, this might have tempered the magnitude of the effects. Fourth, the subjects' expectations regarding the colored cues were not assessed explicitly; so that we cannot exclude that other mechanisms than expectancy effects might be responsible for the placebo and nocebo effects found in this study. Lastly, knowledge of nocebo and placebo effects on itch may, in the long term, help improve therapeutic interventions by reducing unfavorable expectations and enhancing favorable expectations in patients suffering from chronic itch. It remains to be established whether the findings of experimentally induced sensations in healthy subjects can be generalized to patients in a clinical setting. Moreover, the role of individual characteristics in nocebo and placebo responsiveness should be elucidated to further personalize interventions and to optimize treatment outcomes.

In conclusion, this study showed that, in accordance with research on other physical sensations, the combination of conditioning and verbal suggestion can induce significant nocebo and placebo effects on itch. Research on nocebo and placebo effects at a perceptive and neurobiological level is warranted to further elucidate the common and specific mechanisms underlying nocebo and placebo effects on itch and other physical sensations.
